# Markers of Oral Lichen Planus Malignant Transformation

**DOI:** 10.1155/2018/1959506

**Published:** 2018-02-26

**Authors:** Mircea Tampa, Constantin Caruntu, Madalina Mitran, Cristina Mitran, Isabela Sarbu, Laura-Cristina Rusu, Clara Matei, Carolina Constantin, Monica Neagu, Simona-Roxana Georgescu

**Affiliations:** ^1^“Victor Babes” Clinical Hospital for Infectious Diseases, 281 Mihai Bravu, 030303 Bucharest, Romania; ^2^“Carol Davila” University of Medicine and Pharmacy, 37 Dionisie Lupu, 020021 Bucharest, Romania; ^3^Department of Physiology, “Carol Davila” University of Medicine and Pharmacy, 8 Eroii Sanitari, 050474 Bucharest, Romania; ^4^Department of Dermatology, “Prof. N. Paulescu” National Institute of Diabetes, Nutrition and Metabolic Diseases, 22-24 Gr. Manolescu, 011233 Bucharest, Romania; ^5^Oral Pathology Department, Faculty of Dentistry, “Victor Babeș” University of Medicine and Pharmacy, 2 Eftimie Murgu, 300041 Timisoara, Romania; ^6^Immunology Department, “Victor Babes” National Institute of Pathology, 99-101 Spl. Independentei, 050096 Bucharest, Romania; ^7^Colentina Clinical Hospital, 19-21 Ştefan cel Mare Blv., 020125 Bucharest, Romania; ^8^Faculty of Biology, University of Bucharest, 91-95 Spl. Independentei, 76201 Bucharest, Romania

## Abstract

Oral lichen planus (OLP) is a chronic inflammatory disease of unknown etiology with significant impact on patients' quality of life. Malignant transformation into oral squamous cell carcinoma (OSCC) is considered as one of the most serious complications of the disease; nevertheless, controversy still persists. Various factors seem to be involved in the progression of malignant transformation; however, the mechanism of this process is not fully understood yet. Molecular alterations detected in OLP samples might represent useful biomarkers for predicting and monitoring the malignant progression. In this review, we discuss various studies which highlight different molecules as ominous predictors of OLP malignant transformation.

## 1. Introduction

Oral lichen planus (OLP) is a chronic inflammatory T cell-mediated disease, clinically manifested as white, lacy plaques, located mainly on the buccal mucosa and tongue [1, 2]. Several clinical entities of OLP—namely, reticular, atrophic, plaque-like, erosive, and bullous—have been described. Histopathologically, OLP is characterized by the presence of a band-like lymphocytic infiltrate at the interface between the epithelium and connective tissue and by the destruction of the basal layer [1].

According to the World Health Organization, OLP is categorized into the group of potentially malignant disorders [3, 4], while its most severe complication is the progression into oral squamous cell carcinoma (OSCC).

OSCC accounts for 90% of malignant tumors of the head and neck region. It is most commonly diagnosed in 60–70-year-old individuals. It is a multifactorial disease; tumor development is based on both genetic and environmental factors [5, 6]. Various risk factors have been suggested, but controversy still persists. Thus, the main factors proposed to be involved in OSCC pathogenesis include smoking, immunosuppressive agents, chronic inflammation, certain viruses, accumulation of genetic mutations, and a diet low in fresh vegetables and fruits [3, 7–10]. The chosen therapeutic approach for OSCC depends on the stage of the disease and the nonsurgical methods represent an increasingly studied field [11–14].

The first case of malignant transformation of OLP was reported in 1910 [15]. Landini et al. analyzed the studies related to the malignant transformation of OLP during 1924–2012. The reported rate of OLP malignant transformation varied between 0 and 10% [16]. A recent meta-analysis reported that 1.1% of OLP lesions progress into OSCC with a higher incidence in smokers, alcohol users, and in those infected with hepatitis C virus [17]. It seems that erosive OLP is the type that has the highest frequency to progresses into OSCC [18, 19]. Most commonly, malignant transformation occurs in lesions that are localized on the tongue [20]. Muñoz et al. have found that, on the average, it takes 5.5 years for OLP lesions to transform into an established OSCC; moreover, the study has revealed that patients with OSCC developed on a preexisting OLP lesions show a higher rate of tumor recurrence when compared to those with primary OSCC [21].

Current research shows that the malignant process is based on increased proliferation of the basal-layer cells under the influence of mediators released from the inflammatory infiltrate that activate different pathways and can lead to tumor development [22]. Recently, a significant amount of studies aimed at identifying robust biomarkers that can predict the malignant potential of OLP lesions; hence, in this review, we present the latest studies focused on this issue.

## 2. Method

We performed a nonsystematic review using Pubmed and Google Scholar databases. We selected articles written in English based on relevance and our experience in the presented topic; we did not use conventional inclusion and exclusion criteria.

## 3. Results and Discussions

We identified a great number of markers of oral lichen planus malignant transformation and classified them into the following categories: apoptosis-related biomarkers, cell cycle regulators, tissue remodeling factors, inflammation-related factors, galectins, and intercellular adhesion proteins.

### 3.1. Apoptosis-Related Biomarkers

**Figure 1:**
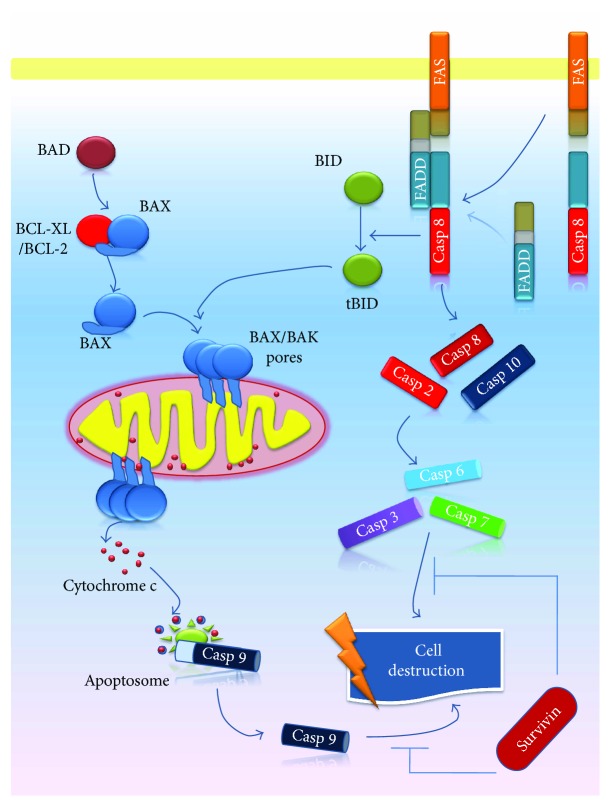
Schematic illustration of apoptosis. Intracellular apoptotic signaling pathways are represented by the intrinsic (mitochondrial) pathway—the activation of BAX and BAK proapoptotic proteins, release of cytochrome c from mitochondria, formation of the apoptosome, and caspase 9 activation—and the extrinsic (death receptor) pathway—FasR/FasL interaction, association of FADD to the complex, followed by DISC formation, and caspase 8 activation. The end point of both intrinsic and extrinsic pathways is cell destruction.

Alterations of apoptosis pathways proved to be involved in general in the onset of malignant processes [23, 24]. Two main pathways of apoptosis have been described, namely, the intrinsic (mitochondrial) and extrinsic (death receptor) pathways (Figure 1) [25]. Certain stimuli, such as hypoxia or free radicals, trigger the initiation of the intrinsic pathway inducing an increased permeability of mitochondrial pores, followed by the release of proapoptotic molecules, such as cytochrome c and apoptosis-inducing factor (AIF), from mitochondria into the cell cytoplasm. Cytochrome c activates procaspase 9, resulting in formation of the apoptosome [26]. This pathway is modulated by B-cell lymphoma protein 2 (BCL-2) family and proteins including proapoptotic proteins (BCL-2-associated X protein (BAX), BCL-2 antagonist killer 1 (BAK), BCL-2 antagonist of cell death (BAD), BH3 interacting-domain death agonist (BID), etc.) and antiapoptotic proteins (BCL-2, BCL-2-related protein (BCL-XL), BCL-2-like 2 protein (BCL-W), myeloid cell leukemia-1 (MCL-1), etc.) [25].

The extrinsic pathway implies the binding of transmembrane receptors (called death receptors), primarily tumor necrosis factor (TNF) receptors and FasR, to their corresponding ligands—TNF-*α* and FasL, respectively [26]. After the connection of ligand to the receptor, the TNF receptor-associated death domain (TRADD) and Fas-associated death domain (FADD) are recruited. The formed complexes associate with procaspase 8, resulting in the formation of death-inducing signaling complex (DISC) and subsequently activation of caspase 8 [27–29]. The end point of both intrinsic and extrinsic pathways is the activation of caspases, leading to cellular destruction [26] (Figure 1).

An increasing number of studies aiming to emphasize the malignant potential of OLP have focused on the evaluation of apoptosis and identification of useful biomarkers [30, 31].

Apoptosis regulation of basal keratinocytes seems to be a key process in the pathogenesis of OLP. Apoptosis can be induced through various mechanisms which activate caspase pathways leading to keratinocyte death. CD8+ cytotoxic T lymphocytes bear a central role in apoptosis promotion (e.g., through secretion of TNF-*α* and release of granzymes). Moreover, it has been shown that a decreased number of apoptotic inflammatory cells in OLP lesions contribute to progression into OSCC [32–34].

Kaur et al. analyzed the apoptotic cells in the saliva of patients with precancerous lesions (OLP, leukoplakia, and oral submucosal fibrosis) and OSCC and found a significantly lower number of salivary apoptotic cells in OSSC compared to that in precancerous lesions [35].

The main proapoptotic molecules are p53, caspase 3, and BAX. Caspase 3 is considered an early marker of apoptosis [36, 37]. In the study by Calenic et al., expression of caspase 3 was lower in OLP than that in the control group, while BAX expression was higher in OLP than that in controls. P53 expression was increased in the OLP group compared to that in controls and BCL-2 expression showed no differences between the two groups. These results may lead to the conclusion that in OLP, an antiapoptotic mechanism is initiated and OLP is resistant to p53-mediated caspase-3-dependent apoptosis [37].

#### 3.1.1. p53

*p53* is the major tumor suppressor gene located on chromosome 17 encoding one of the main proteins, p53 [38], involved in the prevention of carcinogenesis. This protein is involved in DNA repair and destruction of defective cells through the induction of apoptosis. Thus, processes such as cell cycle arrest, apoptosis, and senescence are governed by the activation of p53 [39].

**Figure 2:**
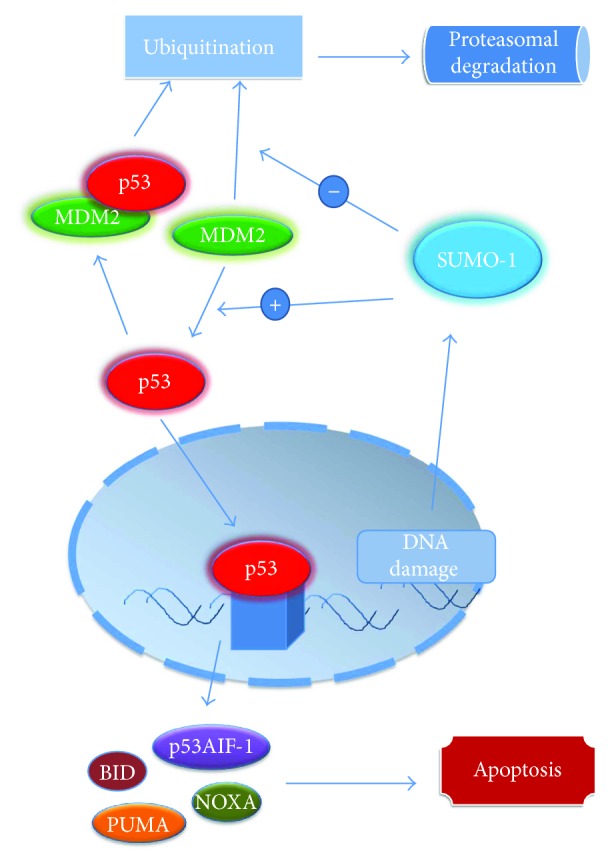
The relationship of p53, MDM2, and SUMO-1 in apoptosis regulation. MDM2 induces p53 ubiquitination and its proteasomal degradation, acting as an E3 ubiquitin ligase; the decrease in p53 level will lead to an inhibition of apoptosis. Under normal conditions, SUMO-1 regulates MDM2 level: MDM2 is undergoing self-ubiquitination and proteasomal degradation; DNA damage leads to SUMO-1 binding MDM2 and blocking its self-ubiquitination, conducting to an increase in MDM2 ubiquitin ligase activity towards p53. While p53 acts by inducing transcription of p53AIF-1, PUMA, BID, and NOXA, leading to the induction of apoptosis, reducing p53 levels by MDM2- and SUMO-1-conjugated action will lead to inhibition of apoptosis.

Under normal conditions, p53 level is low as a result of rapid proteolysis: p53 is inactivated by mouse double minute 2 homolog (MDM2), which enhances the degradation of p53 by proteasomes. In p53-induced apoptosis, mRNA increases for BID [40]; p53 also induces p53 upregulated modulator of apoptosis (PUMA) and NOXA expression, followed by the release of BAX and BAK from their complexes with antiapoptotic proteins and consequently mitochondrial outer membrane channel formation (Figure 2) [41–44]. Moreover, p53 releases BAK from the complex that the latter forms with MCL-1; therefore, BAK protein becomes available for mitochondrial pore formation [45]. Another mechanism by which p53 protein contributes to the initiation and progress of apoptosis is the increase in transcription of p53-regulated apoptosis-inducing factor-1 (p53AIF-1) protein; p53AIF-1 is a protein found in mitochondria, and its action involves the dissipation of the mitochondrial transmembrane potential, an important event of the intrinsic apoptotic pathway followed by the cytosolic release of the cytochrome c and other mitochondrial proapoptotic proteins [43].

Valente et al. evaluated p53 expression in 28 patients diagnosed with OLP. Of those, 15 did not show any degree of dysplasia, 7 presented concurrently OLP and OSCC, and 6 progressed to OSCC. An enhanced expression of p53 protein was observed in patients with OLP and OSCC and in those who progressed to OSCC, as compared to those with OLP without dysplastic lesions. They raised the hypothesis that mutations in the *p53* gene might be involved in malignant transformation and suggested that p53 overexpression might be an indicator of malignant transformation [46]. A recent study also revealed a higher expression of p53 in the saliva of patients with OSCC than in those with OLP [47]. Crosthwaite et al. suggested that benign lesions that are positive for p53 should be carefully monitored [48]. In addition, in the study by Tanda et al., in 20% of OLP cases, the suprabasal layer localization of p53 was observed [49]. Studies have shown that the suprabasal expression of p53 is associated with an increased risk of malignant transformation, such as the study of Cruz et al. that revealed that 86% of premalignant lesions expressing p53 in the suprabasal layers have evolved into carcinomas [50].

p63 and p73 belong to the p53 family, having roles in embryogenesis and cell differentiation. p63 and p73 exhibit functions similar to p53, being involved in the removal of damaged cells and induction of apoptosis. It was observed that when *p53* gene is mutated, *p73* can substitute its function to a certain extent. There are few studies on the role of p73 in OLP [51, 52]. The increased expression of p73 was observed in samples of dysplastic oral mucosa, irrespective of the degree of dysplasia, compared to normal mucosa [53]. *p63* gene encodes proteins with essential role in the development of oral mucosa, salivary glands, teeth, and skin [53, 54]. Ebrahimi et al. detected antibodies against p63 and p73 in sera of OLP patients and speculated that there might be a positive correlation with the duration and severity of the disease [55].

Although one would think that an overexpression of a tumor-suppressor protein would not be an indicator of tumorigenesis, the above-presented findings show that subtle mutations in the gene that encodes for p53 family of proteins can be at the base of antiapoptotic and protumorigenic transformations in OSCC.

#### 3.1.2. MDM2 and SUMO-1

There is a strong link between p53, MDM2, and small ubiquitin-like modifier 1 (SUMO-1), molecules involved in cell proliferation and apoptosis. P53 is inactivated by MDM2 that increases p53 proteasomal degradation. MDM2 acts as an E3 ubiquitin ligase for p53: following ubiquitination under the action of MDM2, p53 will be massively degraded by proteasomes, leading to a decrease in p53 level and, consequently, to the apoptosis inhibition [56]. In addition, it appears that MDM2 can restrain p53 activity by forming a complex with p53 [57].

As for MDM2, its level is regulated by SUMO-1; under normal conditions, MDM2 is undergoing self-ubiquitination and proteasomal degradation; in case of DNA damage, SUMO-1 binds MDM2 and abrogates its self-ubiquitination, leading to an increase in MDM2 ubiquitin ligase activity towards p53. In this particular manner, SUMO-1 regulates MDM2 level and, subsequently, p53 level (Figure 2) [58].

Katayama et al. have revealed that overexpression of MDM2 as an effect of SUMO-1 overexpression may function as a marker of tumor development and aggressiveness even in OSCC's early stages. In this light, SUMO-1 in conjunction with MDM2 might be employed not only as an indicator for tumor occurrence but also as a possible target for future pharmacological therapy [59].

However, another study that has analyzed the expression of proteins p53, MDM2, and SUMO-1 in 4 diseases localized on the oral mucosa (inflammatory fibrous hyperplasia, OLP, oral epithelial dysplasia, and OSCC) compared to normal mucosa proved p53 and MDM2 overexpression in OLP, establishing hence a promalignant transformation environment. As for the expression of SUMO-1 in OLP, it was found to be similar in both normal mucosa and inflammatory fibrous hyperplasia, implying that alterations of SUMO-1 develop at later stages of carcinogenesis, as an important overexpression of this protein was found in oral epithelial dysplasia and established OSCC [60].

#### 3.1.3. BCL-2/BAX

BCL-2 is an inhibitor of apoptosis, whereas BAX, also a member of BCL-2 family, participates in the activation of intrinsic apoptotic pathway, having an opposite effect to BCL-2. Elevated expression of BCL-2 promotes a high survival rate of malignant cells that predisposes to an increased risk of developing new mutations [30, 36, 61].

An increased expression of BCL-2 was found in the lymphocytic infiltrate which is characteristically encountered in OLP lesions, on one hand. On the other hand, the expression of BAX was elevated in epithelial basement keratin in OLP group compared to that in the control group, which included healthy subjects. These observations suggest a link between alteration of apoptosis and carcinogenesis [62]. The same idea is supported by the study of Pigatti, which found that 92% of OLP patients had a positive expression of BCL-2 in inflammatory infiltrate [63]. It seems that BCL-2 plays a role in the inhibition of apoptosis of lymphocytes, while BAX is involved in the induction of apoptosis of keratinocytes [64]. Although scientifically tempting to consider BCL-2 as a prognostic marker, Hadzi has postulated that BCL-2 should not be regarded as a prognostic marker for OSCC development [65].

#### 3.1.4. MCL-1

**Figure 3:**
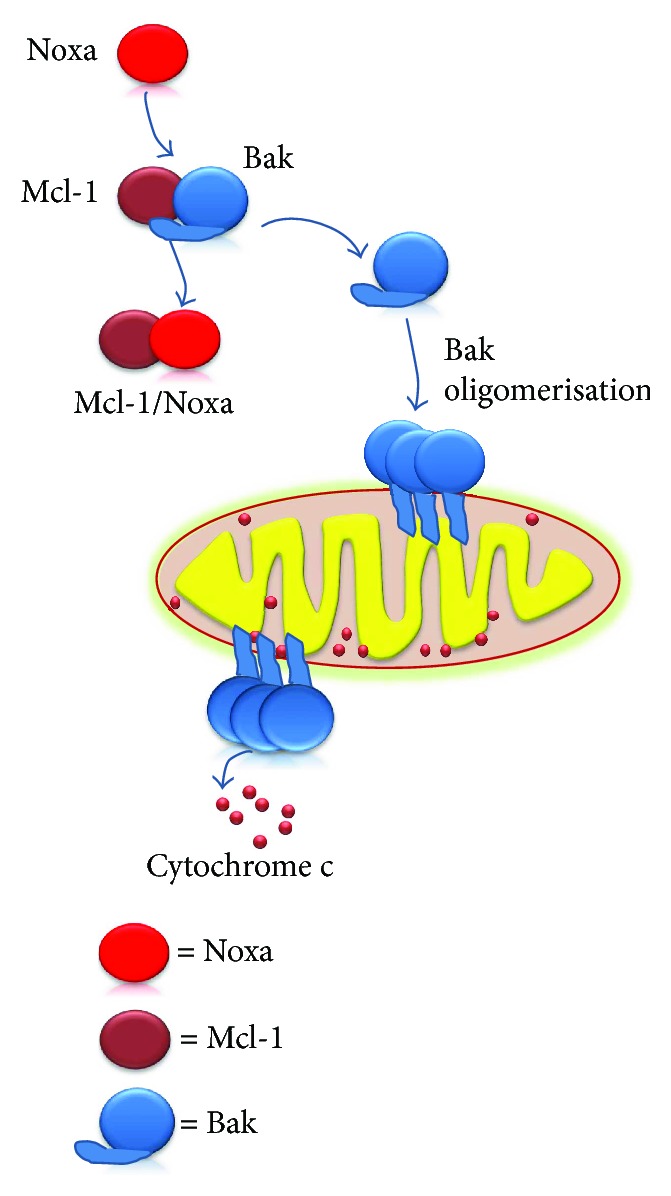
The role of MCL-1 in apoptosis. Under normal conditions, MCL-1 binds BAK. As a result of various cytotoxic signals, NOXA can displace BAK from this bondage. Subsequently, BAK leads to channel formation in mitochondria, cytochrome c release, and caspase activation.

MCL-1 is an antiapoptotic protein pertaining to the BCL-2 family, it binds proapoptotic protein BAK in normal healthy cells. BAK is therefore sequestered until various cytotoxic signals activate a combination of BH3-only proteins that can displace BAK from this bondage, such as NOXA. Consequently, BAK can form oligomers that will organize as channels in mitochondria, leading to cytochrome c exiting into the cytosol and, subsequently, to caspase activation and apoptosis execution (Figure 3) [66].

Shin et al. suggested that MCL-1 could be a novel biomarker for the malignant potential of OLP. However, the study included a small number of samples (11 biopsies of OLP, 3 of normal human oral mucosa, and 2 human oral cancer cell lines—MC-3 and HSC-3). Expression of MCL-1 was increased in OLP lesions and in the 2 tested cell lines, compared to that in the normal mucosa. The study highlighted the decrease of MCL-1 expression in cancer lines after treatment with sorafenib and mithramycin A, along with a decrease in cell replication, suggesting that the decline of MCL-1 expression could influence the process of malignant transformation [67].

In many neoplasms, it was observed that MCL-1 overexpression is associated with cell resistance to apoptosis. This phenomenon is based on the interaction between MCL-1 and proapoptotic members of the BCL-2 family (BAK, BAX, etc). In another study on OSCC, Shin et al. evaluated the role of mithramycin and it was found that it inhibits MCL-1 expression. Using this chemotherapeutic agent, good therapeutic results were obtained, therefore supporting once more the role of MCL-1 in carcinogenesis [68].

#### 3.1.5. Survivin

Survivin is a member of the inhibitor of apoptosis (IAP) gene family. It seems that survivin plays an important role in carcinogenesis, being a molecule that modulates apoptosis and inhibits cell division [69]. Multiple molecular mechanisms for survivin involvement in carcinogenesis have been shown; one of them is survivin's inhibitory action of caspase 9, caspases 3 and 7, inhibition that leads to apoptosis blocking (Figure 1). Moreover, survivin expression seems to be modulated, among others, by p53 protein: wild-type p53 represses survivin transcription, an effect that mutated p53 seems to fail to accomplish [70]. In contrast, survivin enhances p53 proteasomal degradation as a result of inhibition of MDM2 cleavage by blocking the caspases [71].

A recent study investigated the role of survivin in OLP. Survivin expression with moderate intensity was observed in the basal layer of OLP lesions. Its expression in normal tissues was modest or absent, while a higher expression of survivin was identified in OSCC samples, suggestive for its role in carcinogenesis [72].

### 3.2. Cell Cycle Regulators

Deregulators of cell cycle controllers have also been studied as a possible important process involved in the malignant transformation of OLP. Most studies focus on the role of p16, B-cell-specific-Moloney murine leukemia virus integration site 1 (BMI1), and Ki67.

#### 3.2.1. p16 and Cyclin-Dependent Kinases

Cell cycle is governed by the action of cyclin-dependent kinases (CDKs) and their main inhibitors p16, p21, and p27, which are important tumor suppressors [73]. CDKs associate with cyclin proteins and act on the cell cycle. CDK4 and CDK6 associate with cyclin D and participate in G1 phase progression. Cyclin D, CDK4, and CDK6 form a complex that promotes the progression of cells from S to G1 phase by phosphorylation of retinoblastoma proteins (pRb). p16 has an inhibitory role in cyclin D-CDK4-CDK6 complex, preventing phosphorylation of pRb, and the final result is the inhibition of the cell cycle [74].

As aforementioned, p16 protein is involved in the antitumor response, promotes tumor suppression, and acts on the cell cycle [75]. Increased levels of p16 have been identified in senescent cells; therefore, it has been suggested that p16 may be responsible for the induction of cell senescence, preventing malignant cell transformation. Loss of p16 expression is a feature commonly found in neoplasms and has been early detected in the process of carcinogenesis [76]. Montebugnoli et al. analyzed the role of p16 in the progression of OLP into OSCC. They found an increased expression of p16 in 64% of OLP patients as compared to that in only 28% of patients with oral leukoplakia. No differences were observed between samples from patients with OLP and those with nonspecific reactive inflammation. Interestingly, differences were observed between leukoplakia with signs of inflammation, where p16 expression was increased, and leukoplakia without signs of inflammation, where p16 expression was normal [77]. The findings were in line with other studies that have shown a link between proinflammatory cytokines such as TNF-*α* and an increased p16 expression [78]. Montebugnoli concluded that p16 expression is influenced by the presence of inflammation, being overexpressed in such conditions, and the results should be interpreted taking into account that particular fact [77]. Moreover, Salehinejad et al., also studying p16 expression in OLP patients, concluded that the effect of cytokines on p16 expression should not be neglected; therefore, it cannot be used as a predictor of malignant transformation [79]. However, in other studies, an increased expression of p16 has been identified in 15 to 30% of cases of OSCC [80–82].

Goel et al. have revealed an increased expression of cytoplasmic p16 and CDK4 in OLP patients compared to that in normal mucosa. However, compared to OSCC, cytoplasmic expression of p16 and CDK4 were lower in OLP. Comparing nonerosive and erosive OLP, only the overexpression of cytoplasmic CDK4 was observed in erosive OLP samples. Cytoplasmic expression of p16 and CDK4 might be a predictor of the OLP malignant progression [83]. In addition, Poomsawat et al. studied the role of CDK6, but he noticed that CDK6 expression in OLP did not differ from normal mucosa, an observation which suggests that CDK6 is not significantly involved in OLP pathogenesis [74].

#### 3.2.2. BMI1

BMI1, a polycomb-group protein and a stem cell factor, is involved in cell cycle and in cell proliferation and plays a role in the self-renewal of stem cells. Increased expression of BMI1 has been determined in many tumors [84] and seems to be associated with cellular dysplasia, a process on which carcinogenesis is based [85].

BMI1 was proposed by Ma et al. as a marker for identifying oral lesions at high risk of progressing into OSCC [86]. After taking samples from 96 patients with OLP, followed over a period of 54 months, the authors identified 87 patients with OLP who did not evolve into OSCC and 9 patients with OLP who did. In the first group, BMI1 expression was identified in 36.8% of cases (32 out of 87), while in the second group in 88.9% (8 out of 9) of cases. They also analyzed the BMI1 expression in 10 patients with normal oral mucosa and 6 patients with OSCC, developed on OLP lesions. None of the samples of normal oral mucosa exhibited BMI1 expression, but BMI1 expression was shown in all OSCC samples [86]. The same authors have found an abnormal expression of BMI1 in oral samples of leukoplakia [87].

Other research has investigated the possible links between BMI1 and p16 functions, as previously discussed in this section. Thus, Huber et al. studied 252 samples of oral and oropharyngeal SCC. They found an increased BMI1 expression along with a decreased p16 expression in SCC cells. Moreover, they obtained correlations of these markers with survival and recurrence rates. Thus elevated BMI1 expression and reduced p16 expression correlate with a poor prognosis and a high rate of relapse. It seems that BMI1 has an inhibitory effect on p16 [88].

Another study by Kang et al. brings other important information to our attention regarding BMI1 involvement. The authors blocked endogenous BMI1 in cultures of normal keratinocytes and SCC keratinocytes and found that cell replication was affected suggesting its role in cancer cells proliferation. In addition, the study offers information about the link between BMI1 and p16. Differences in p16 expression in cells with and without BMI1 suppression were not observed and it was suggested that BMI1 may act through p16-independent pathways to stimulate the malignant proliferation process. Moreover, BMI1 overexpression was observed in pre-neoplastic oral lesions, showing various degrees of dysplasia, an important clue that suggests that BMI1 is expressed early in the process of carcinogenesis [85].

#### 3.2.3. Ki67

It is commonly known that Ki67 is involved in the active phases of cell cycle and is considered an ubiquitary marker of cell proliferation. Ki67 is being expressed starting with S phase of the cell cycle and it reaches a maximum when mitosis occurs [38]. Zargaran et al. determined the expression of Ki67 in patients with epithelial hyperplasia, in patients with OLP, in patients with varying degrees of epithelial dysplasia of oral mucosa, and in patients with well or poorly differentiated OSCC. Ki67 expression progressively increased from epithelial hyperplasia to OSCC. Ki67 expression in OLP was higher than that in epithelial hyperplasia but similar to that in mild dysplasia. The number of cells with genetic alterations was higher in OLP than that in epithelial hyperplasia [89]. Studies have shown that there is a link between Ki67 expression and loss of heterozygosity [77].

### 3.3. Tissue Remodeling Factors—Matrix Metalloproteinases and Their Inhibitors

Matrix metalloproteinases (MMPs) are zinc-dependent enzymes involved in inflammatory and malignant processes [6, 90–93]. Under the action of factors such as transforming growth factor beta (TGF-*β*) and interleukin 8 (IL-8), macrophages, neutrophils, and fibroblasts release various MMPs. MMPs participate in the process of malignant transformation through mechanisms such as stimulation of different growth factors or inhibition of natural killer (NK) cell function. Additionally, MMPs regulate the bioavailability of vascular endothelial growth factor receptor (VEGFR) and consequently promote angiogenesis [94, 95].

Giannelli et al. conducted the first study that revealed the role of MMPs in the pathogenesis of OLP and hypothesized that these molecules may be involved in the destruction process of the basement membrane through an imbalance between the level of MMPs and their inhibitors [96].

Other research that included patients with oral leukoplakia showed a positive correlation between MMP-9, VEGFR2, and the degree of epithelial dysplasia [97].

Chen et al. investigated the expression of MMPs (MMP-2, MMP-9) in normal mucosa, nonatrophic OLP, atrophic OLP, and OSCC, and they observed a progressive increase of their expression. The same progressive increase was observed for tissue inhibitor of metalloproteinases (TIMP) 2, an inhibitor of MMP-2, and TGF-*β*1, a modulator of the MMPs activity. The study suggested that MMPs, especially MMP-9, might be a predictor of OLP lesions' malignant transformation. This research showed that atrophic OLP apparently has a higher risk of malignant progression than nonatrophic OLP [98]. In addition, it has been shown that MMP-9 expression is increased in the tissue, saliva, and serum of patients with premalignant oral lesions (OLP, oral leukoplakia, and oral submucous fibrosis) compared to that of the healthy individuals [94]. Immunohistochemistry studies on OLP specimens have revealed expression of MMP-2 and MMP-3 in the epithelium, while MMP-9 was identified in the adjacent inflammatory infiltrate [99, 100]. Agha-Hosseini et al. have noticed that MMP-3 expression has increased gradually when they analyzed cases of reticular OLP, erosive OLP, early-stage OSCC, and advanced OSCC [101].

The physiological inhibitors of MMPs are TIMPs. Shrestha et al. have studied the complex MMP-2/TIMP 2 in OSCC lesions and revealed that the activity of MMP-2 might be a marker associated with a low survival rate. The two markers correlated with the stage of the disease and the existence of metastases [102]. In addition, in accordance with the study by Katayama et al., TIMP 2 is an important marker which may be used to identify patients with SCC with poor prognosis in early stages of evolution [103]. Another study revealed that an increased level of MMP-13 could be used as a biomarker in OSCC of the tongue [90]. However, Agha-Hosseini et al. observed no differences in saliva and serum levels of MMP-13 between patients with OLP and OSCC [104].

### 3.4. Inflammation-Related Factors

#### 3.4.1. Cytokines

Inflammatory cells release a variety of molecules that may be involved in cell proliferation and angiogenesis. Studies have shown that in the course of a chronic inflammatory process, cytokines can participate in malignant cell transformation, contributing to an increase in mutation rate [105–107]. Cytokines and chemokines encountered in tumor microenvironment have a pivotal role in tumor progression, exhibiting an inhibitory or stimulatory effect. Thus, interleukins such as IL-6, IL-17, or IL-23 contribute to tumor progression, and TNF-*α*, TGF-*β*, or IL-6 has a direct effect on the cell growth and survival rate [108].

It seems that inflammatory processes may play a role in the progression of OLP lesions into OSCC [109]. Rhodus et al. proposed the determination of salivary cytokines as a method of monitoring the evolution of OLP. They determined the level of TNF-*α*, IL-1, IL-6, and IL-8 (NF-κB-dependent cytokines) in the saliva of 13 OLP patients with different degrees of dysplasia, 13 OSCC patients and 13 control subjects. TNF-*α* levels were elevated in OLP patients with moderate or severe dysplasia, similar to those with OSCC, while IL-6 and IL-8 levels were lower in OSCC. IL-1a level in moderate dysplasia was similar to that identified in OSCC samples, whereas in severe dysplasia, it was lower [110]. Similar results were obtained by Juretic et al., and they proposed TNF-*α* and IL-6 as markers with prognostic significance [111]. Lisa Cheng et al. also highlighted that IL-6 measurement in saliva can be a useful tool in OSCC detection [112]. Another study has revealed that IL-10 is increased in early stages of leukoplakia and OSCC, whereas interferon gamma (IFN-*δ*) is decreased in advanced cases of leukoplakia and OSCC [109].

Like in other types of cancer, the proinflammatory cytokine types prevail upon the anti-inflammatory ones sustaining thus the tumorigenesis potential of inflammation.

#### 3.4.2. Cyclooxygenase-2

As previously discussed, the presence of inflammation, especially chronic inflammation, is a condition contributing to the occurrence of carcinogenesis. Inflammatory stimuli lead to the activation of numerous signaling pathways, including cyclooxygenase (COX) expression [113]. COX has two isoforms: COX-1 and COX-2. COX-1 is expressed in normal tissues and plays a role in the maintenance of homeostasis, while COX-2 expression is induced by inflammatory molecules, growth factors, or hormones. Under normal conditions, COX-2 is almost not expressed in most tissues [113, 114]. Increased expression of COX-2 has been identified in many cancers (e.g., gastric cancer and lung cancer) [115]. Recent studies have shown that COX-2 participates in the process of carcinogenesis by inhibiting apoptosis, stimulating angiogenesis, and inducing immunosuppression [116]. Based on these observations, COX-2 is considered a prognostic marker in various malignancies [117].

Changkong reported an increased COX-2 expression in OLP lesions and the correlation between COX-2 expression and disease severity [118]. The study conducted by Neppelberg and Johannessen concluded that COX-2 is not a marker for malignant transformation of OLP into SCC [119]. However, studies suggest that COX-2 may represent a marker indicating the risk of malignant transformation of precancerous oral lesions [115, 120]. Itoh et al. have showed overexpression of COX-2 in 13.9% of SCC cases. They established that COX-2 overexpression correlates with lymph node involvement, tumor recurrences, and disease-free survival. They have highlighted the role of COX-2 inhibitors in SCC therapy [121].

### 3.5. Galectins—Important Players in Inflammation and Carcinogenesis

Galectins comprise a family of endogenous carbohydrate-binding proteins with affinity for b-galactosides [122]. Galectins act on immune cellular processes through intra- and extracellular mechanisms, such as the stimulation of inflammation, activation of T cells, and modulation of Treg cell activity. Galectins are involved in growth, migration, adhesion, and cell apoptosis. Galectins participate both in immunomodulation and stimulation of angiogenesis. Galectin 1 confers an immune protection to malignant cells, allowing them to avoid host immune response [123–125]. Galectin 9 has a dual action; it promotes T cell death via the C terminal domain on the one hand and leads to proliferation and activation of dendritic cells via the N terminal domain [126] on the other hand. It seems that galectin 9 is involved in CD3 and CD8 T cell death by activating caspase 1 [127].

Muniz et al. emphasized the role of galectins in the differentiation of OSCC and premalignant lesions. They studied the expression of galectins 1, 3, and 9 in OSCC, OLP, and oral leukoplakia in comparison with a normal histopathological profile. They have noticed a higher expression of galectin 9 in OSCC samples compared to that in samples of oral premalignant conditions and samples with normal histopathological aspect. The results regarding the expression of galectin 1 and 3 were heterogeneous in the studied groups [127].

Ding et al. proposed galectin 1 as a predictor for progression of oral leucoplakia lesions into OSCC, revealing the overexpression of this protein in biopsies taken from patients with OSCC and oral leukoplakia [128]. Galectin 1 overexpression was identified in several types of cancer including melanoma, prostate cancer, or laryngeal SCC [129, 130]. Noda et al. have shown that galectin 1 could be a useful marker in classifying lesions of the mouth in reactive and neoplastic lesions [131].

### 3.6. Intercellular Adhesion Proteins—E-Cadherin Role

Transmembrane cadherins are proteins involved in intercellular adhesion and cellular differentiation. Reduction in their expression is associated with loss of characteristics of epithelial cells, and therefore, cadherins are involved in tumor differentiation, lymph nodes invasion, and occurrence of metastases. Altered E-cadherin expression was reported in large, poorly differentiated and metastatic tumors, E-cadherin being known as a molecule which inhibits tumor progression [132, 133]. Regarding OLP, the E-cadherin expression results are highly contradictory.

Du and Li have revealed abnormal positive expression of E-cadherin among OLP patients. Thus, 51.9% of the OLP patients had an abnormal positive expression of E-cadherin compared to only 4.8% of healthy controls. Based on the results of various studies in which E-cadherin expression was associated with the development of malignancies, it has been suggested that E-cadherin may be a marker of malignant transformation of OLP [134]. Sridevi et al. analyzed the expression of E-cadherin in several diseases of the oral cavity, oral submucosal fibrosis, oral leukoplakia, OLP, and OSCC. In the OLP group, E-cadherin expression was weak in six subjects and moderate to strong in three subjects; in the OSCC group, the results were similar. Low E-cadherin expression was associated with poorly differentiated cancers. These results do not allow the conclusion that E-cadherin is a prognostic marker of the malignant transformation of OLP [135]. Moreover, Neppelberg and Johannessen found that there is no correlation between the loss of E-cadherin expression and the risk of the malignant development of OLP lesions [119]. However, in another study, they concluded that E-cadherin may be involved in the destruction of the basal layer and T cell migration into the epithelial compartment in OLP lesions [136].

## 4. Conclusions

OLP is considered by many researchers as a premalignant lesion; therefore, patients should be monitored in order to identify ominous signs of malignant transformation into OSCC in the very early stages. It is of paramount importance to understand the pathogenesis of OLP and establish what determines its progression to OSCC, as well as to monitor it adequately. Up to now, numerous biomarkers show promising results, including modulators of apoptosis (p53, MCL-1), cell cycle regulators (BMI1, p16), tissue remodeling factors (MMPs), and inflammation-related factors (TNF-*α*, IL-6, and COX-2). Although many researchers have suggested various biomarkers that may be useful in the detection of malignant progression, further studies are needed in order to establish the role of these biomarkers in current medical practice.
